# Preparation and properties of chitosan‐based microspheres by spray drying

**DOI:** 10.1002/fsn3.1479

**Published:** 2020-02-28

**Authors:** Zeng‐liang Zhang, Li‐jie Li, Dan Sun, Min Wang, Ju‐ran Shi, Di Yang, Lu‐hui Wang, Sheng‐can Zou

**Affiliations:** ^1^ R&D Center Yihai Industry Holding Co., Ltd. Qingdao China

**Keywords:** *Atractylodes*, chitosan, *Codonopsis*, microspheres, *Panax notoginseng*, spray drying, sustained release

## Abstract

In this study, the chitosan‐based release microspheres were prepared by spray drying method. Chitosan was used as the carrier material, and *Panax notoginseng* extract, *Codonopsis* extract, and *Atractylodes* extract (the mass ratio was 2:7:5) were active substance. The spray drying preparation process of microsphere was optimized by single factor experiment and L_9_ (3^4^) orthogonal design. Drug loading (DL), particle size, and sustained release performance of microspheres were investigated. The mass fraction of chitosan was 1.5%, the mass ratio of drug to chitosan was 1:3, the inlet air temperature was 130°C, and the injection rate was 400 ml/hr. The chitosan‐based microspheres prepared under the above conditions had a smooth surface, and the DL was 23.87 ± 0.93%; the average particle diameter was 10.27 ± 1.05 μm, and the encapsulation efficiency (EE) of the microspheres was 91.28 ± 1.04%. The preparation process of chitosan‐based drug microsphere prepared by spray drying method was simple and stable. The prepared microspheres in this paper showed a sustained release effect in vitro.

## Graphical abstract

To study the characteristics and the drug release of the chitosan‐based microspheres in vitro. The gastric mucosal protective drug microspheres were used chitosan as the carrier material and prepared with *Panax notoginseng* extract, *Codonopsis* extract, and *Atractylodes* extract by spray drying. The preparation process of chitosan‐based gastric mucosa protective drug microsphere was simple and stable.

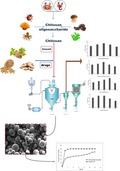

## 1 INTRODUCTION

*Panax notoginseng* (Burk.) is the dried root and rhizome of araliaceae. Its main component is *P. notoginseng* saponins, which have abilities of resisting inflammation, resisting oxidation, lowering blood pressure and blood fat, protecting liver, and improving myocardial ischemia. Modern pharmacological studies have shown that *P. notoginseng* saponins can significantly reduce the stress injury of gastric mucosa and also promote the transformation of gastric mucosal cells from an activated state to a resting state. These indicated that *P. notoginseng* saponins have protective effect on gastric mucosa of stressed rats. It may exert its anti‐stress gastric mucosal damage by regulating the acid secretion function of parietal cells (Deng, Li, & Wang, 2008; Zhang et al., 2013). *Codonopsis* is the dry root of *Codonopsis pilosula*. *Codonopsis* has the effects on reconciling the spleen and stomach, replenishing kidney essence, and improving microcirculation. Modern pharmacological studies have proven that *Codonopsis* plays an important role in regulating gastric contraction and anti‐ulcer (Li et al., 2017; Ma, Shen, Shi, Liang, & Xu, 2013). *Atractylodes*, which has the functions of strengthening the spleen, eliminating moisture, and facilitating urination, is the dry root of *Atractylodes macrocephala* (*Atractylodes macrocephala* Koidz). Modern pharmacological research found that *Atractylodes* can treat spleen and stomach diseases, and its pharmacological mechanism mainly lies in two aspects, namely regulation of gastrointestinal motility and protection of gastric mucosal (Bose, Han, Lee, & Kim, 2013; Yu et al., 2015). Therefore, this study thoroughly mixed the *P. notoginseng* extract, *Codonopsis* extract, and *Atractylodes* extract in proportion (2:7:5) as a gastric mucosal protective drug (Hereinafter referred to as “drug”).

Traditional drug administration methods often cause blood drug concentration to fluctuate greatly in a short period of time. However, excessive blood concentration will cause side effects, and then the blood concentration will decrease rapidly, which may lead to inadequate treatment and decreased drug efficacy. Therefore, maintaining proper blood concentration and residence time is extremely important for treatment (Ding, Huang, Li, & Liu, 2007). On the one hand, the sustained release technology can prolong the drug treating time, so that the drugs can exert the best effect in the human body meanwhile reducing side effects; on the other hand, the preparation of the microspheres can mask the bad smell of the drugs. In this study, chitosan was used as a carrier to prepare drug microspheres. Chitosan is a safe and non‐toxic natural polymer polymeric amino polysaccharide. It contains many amino and hydroxyl groups. It has good water absorption capacity and water retention properties. It has good biocompatibility and can be biodegraded by various enzymes such as pepsin and lysozyme. These characteristics make chitosan to be an ideal drug release material (Hejazi & Amiji, 2002, 2004).

Spray drying is a relatively early and practical method for preparing microspheres. First, the capsule core substance is dispersed in a solution of the encapsulating material that has been liquefied in advance, and then the mixed solution is atomized in a hot air flow to rapidly evaporate the solvent of the solution encapsulating material. As a result, the capsule membrane is cured, and the encapsulated core material is microspheroidized. This method has the advantages of easy to model, short drying time, and good product quality and easy to industrialization produce (Hong, Wang, & Wu, 2000). In recent years, spray drying technology has been widely used in food, pharmaceutical, and feed industries (Gavini et al., 2009; Umurönal, 2009). Used hydrolyzed gelatin as the wall material and lycopene crystals as the core material, lycopene microcapsules prepared by spray drying method had significantly improved stability compared with lycopene crystals (Xu, Jia, Yang, & Lian, 2017). Ginger oleoresin has high viscosity, poor fluidity, and is insoluble in water. There are many constraints on direct use. Using microcapsule technology to turn ginger oleoresin into a solid powder can protect aromatic substances from external factors and effectively control the release of scent substances (Gu, Song, & Wang, 2009).

In this study, chitosan was used as the carrier material, and the chitosan‐based gastric mucosal protective agent microspheres were prepared by spray drying. The total saponins of *P. notoginseng* were used as the detection index. The microsphere yield (YD) and encapsulation efficiency (EE) were used as comprehensive evaluation indexes. The spray drying preparation process of microsphere was optimized by single factor experiment and L_9_ (3^4^) orthogonal design. Drug loading (DL), particle size, and sustained release performance of microspheres were investigated.

## 2 MATERIALS AND METHODS

### 2.1 Materials

Ginsenoside Rg1 standard (content ≥ 98%), Ginsenoside Rb1 standard (content ≥ 98%), and Notoginsenoside R1 standard (content ≥ 98%) were purchased from China Standard Material Network. *Panax notoginseng* extract, *Codonopsis* extract, and *Atractylodes* extract were purchased from Xi'an Tianbao Biological Technology Co., Ltd. Chitosan (deacetylation degree ≥ 90%, Xi'an Wanzi Biological Technology Co., Ltd.); Acetonitrile (chromatographic grade, Shanghai Jiu Shui Chemical Co., Ltd.); Glacial acetic acid (analytical grade, Jinan Xinlonghai Industry and Trade Co., Ltd.). The other reagents were analytical grade.

### 2.2 Instruments

SP‐1500 Laboratory Spray Dryer (Shanghai Shunyi Experimental Equipment Co., Ltd.); Agilent 1260 High Performance Liquid Chromatography (Agilent Technologies, Inc.); Kc‐180w Ultrasonic cleaning instrument (Jining Keyuan Ultrasonic Equipment Co., Ltd.); AB105 electronic analytical balance (Shanghai Precision Instrument Co., Ltd.); KYKY‐EM6200 scanning electron microscope (SEM, Beijing Zhongke Technological Development Co., Ltd.); BT‐9300H Laser Particle Size Analyzer (Dandong Baxter Instrument Co., Ltd.); and ZRS‐8G intelligent dissolution tester (Tianjin Tianda Tianfa Technology Co., Ltd.).

### 2.3 Preparation of chitosan‐based microspheres

The chitosan weighed accurately was dissolved in 1% (v/v) acetic acid solution and configured to be used at different mass concentrations. The uniformly mixed drug was accurately weighed and dissolved in 100 ml of chitosan solution, and then, the mixture was thoroughly stirred, mixed uniformly, and ultrasounded at 120 W for 5 min. Chitosan‐based microspheres were obtained by spray drying.

### 2.4 Determination of detection index

In this study, the simulated drugs were uniformly mixed with *P. notoginseng* extract, *Codonopsis* extract, and *Atractylodes* extract (the mass ratio was 2:7:5). The total saponins of *P. notoginseng* were used as the detection index of the study. The formula for calculating the drug quality was as follows:

$$\text{Drug quality}=\frac{L/M}{N}$$

Remark: *L* is the detection content of total saponins of *P. notoginseng*; *M* is the total saponins content of *P. notoginseng* in the extract of *P. notoginseng* (80%); *N* is the percentage of the extract of *P. notoginseng* in the drug content (14.3%).

### 2.5 Establishment of determination method for total saponins of *P. notoginseng*

#### 2.5.1 Chromatographic conditions

Sepax Bio‐C18 column (4.6 mm × 250 mm, 5 μm); Mphase A was acetonitrile, Mphase B was water; flow rate was 1.0 ml/min; detection wavelength was 203 nm; column temperature was 25°C. Injection volume was 10 μl. The elution conditions were as follows: 0–12 min, acetonitrile accounted for 19%, 12–60 min, and acetonitrile increased linearly from 19% to 36%.

#### 2.5.2 Establishment of a standard curve

Ginsenoside Rg1 1.28 mg, ginsenoside Rb1 1.37 mg, and notoginsenoside R1 1.61 mg were weighed accurately in a 10 ml volumetric flask, dissolved in methanol, and set to different concentrations. The concentration of the reference solution was set to 128, 137, and 161 μg/ml, respectively. About 0.25, 0.50, 0.75, 1, 1.5, and 2 ml series of reference solution were accurately drawn, respectively, added in a 10 ml volumetric flask and made up to volume with methanol solution, then filtered through 0.22 μm microporous membrane. Linear regression was performed with peak area (A) and three saponin mass concentrations (ρ). The equation is shown in Table 1. It was indicated that the mass concentrations of ginsenoside Rg1, ginsenoside Rb1, and notoginsenoside R1 were linearly related to the peak area in the linear range as follows.

**Table 1:** Linear regression equations and correlation coefficients

Compound	Linear regression equation	Correlation coefficient (*r* ^2^)	Linear range (μg)
Ginsenoside Rg1	*Y*1 = 1.6142*X* − 0.3827	.9997	3.2–25.6
Ginsenoside Rb1	*Y*2 = 1.3998*X* − 0.2308	.9995	3.425–27.4
Notoginsenoside R1	*Y*3 = 1.7009*X* − 0.2167	.9998	4.025–32.3

#### 2.5.3 Precision test

The ginsenoside Rg1 reference solutions with mass concentrations of 3.2, 12.8, and 25.6 μg/ml were prepared and repeated three times in 1 day. The RSD for 1 day was calculated to be 0.95%, 0.77%, and 0.82%, respectively. At the same time, the RSD on days 1, 2, and 3 was calculated to be 1.13%, 0.89%, and 0.92%, respectively.

The ginsenoside Rb1 reference solutions with mass concentrations of 3.425, 13.7, and 27.4 μg/ml were prepared and repeated three times in 1 day. The RSD for 1 day was calculated to be 0.90%, 0.71%, and 0.88%, respectively. At the same time, the RSD on days 1, 2, and 3 was calculated to be 1.09%, 0.81%, and 0.97%, respectively.

The notoginsenoside R1 reference solutions with mass concentrations of 4.025, 16.1, and 32.3 μg/ml were prepared and repeated three times in 1 day. The RSD for 1 day was calculated to be 0.98%, 0.75%, and 0.84%, respectively. At the same time, the RSD on days 1, 2, and 3 was calculated to be 1.17%, 0.86%, and 0.88%, respectively.

Since the drug was mixed evenly in advance, it could be further verified by precision test. Therefore, the reference substance of notoginsenoside R1 was taken as an example in subsequent tests.

#### 2.5.4 Recovery rate determination

The control substance of notoginsenoside R1 was taken as an example. The accurately weighed notoginsenoside R1 reference 1.61 mg was proportionally added to the blank microspheres, and then the mixture was dissolved in 1% (v/v) acetic acid solution and prepared into a solution with mass concentrations of 4.025, 16.1, and 32.3 μg/ml, respectively. The recovery rates of high, medium, and low mass concentrations were 99.87%, 101.99%, and 99.16%, respectively, and the RSD was 0.67%, 0.80%, and 0.53%, respectively.

#### 2.5.5 Determination of drug loading and encapsulation efficiency of microspheres

About 50 mg microsphere powder was accurately weighed and put into a 25 ml volumetric flask. Acetic acid was added to dissolve and fix the volume. After ultrasound, the powder was fully shaken. About 1.0 ml of the above solution was accurately pipetted into a 10 ml volumetric flask and diluted to the mark with 1% (v/v) acetic acid solution. The peak area (A) at 203 nm was measured and substituted to the regression equation (Table 1) to calculate the mass of notoginsenoside R1 in the microspheres. The drug loading and encapsulation efficiency of the microspheres were calculated according to the following formulas:

$$\text{Drug loading}\;\left(\text{DL}\right)=\frac{\text{Drug content in microspheres}}{\text{Total mass of microspheres}}\times 100\%$$

$$\text{Encapsulation efficiency (EE)}=\frac{\text{Actual drug mass fraction of microspheres}}{\text{Theoretical mass fraction of microspheres}}\times 100\%$$

#### 2.5.6 Determination of yield

The spray‐dried microspheres were collected and accurately weighed, and then the percentage of the feed amount was calculated, which was the yield and calculated as follows:

$$\text{Yield (YD})=\frac{\;\text{Mass of microspheres obtained}}{\;\text{Total amount of feed}}\times 100\%$$

### 2.6 Single factor experiments of microsphere preparation process

The purpose was to investigate the effects of various factors on the preparation of chitosan microspheres. The “(EE + YD)/2” was taken as the comprehensive evaluation index. Single factor experiments were performed on chitosan mass fraction, mass ratio of drug to chitosan, inlet air temperature, and injection rate.

#### 2.6.1 Effect of chitosan mass fraction

The mass ratio of the drug to chitosan was 1:3, the inlet air temperature was 130°C, and the injection rate was 400 ml/hr, which was repeated three times. The effect of different chitosan mass fractions (0.5%, 1%, 1.5%, 2%, and 2.5%) on the preparation of microspheres was investigated.

#### 2.6.2 Effect of mass ratio of drug to chitosan

The chitosan mass fraction was 1.5%, the inlet air temperature was 130°C, and the injection rate was 400 ml/hr, which was repeated three times. The effects of different ratios of drug to chitosan (1:1, 1:2, 1:3, 1:4, and 1:5) on the preparation of microspheres were investigated.

#### 2.6.3 Influence of inlet air temperature

The chitosan mass fraction was 1.5%, the mass ratio of drug to chitosan was 1:3, and the injection rate was 400 ml/hr, which was repeated three times. The effects of different inlet air temperatures (100, 110, 120, 130, and 140°C) on the preparation of microspheres were investigated.

#### 2.6.4 Effect of injection rate

The chitosan mass fraction was 1.5%, the mass ratio of drug to chitosan was 1:3, and the inlet air temperature was 130°C, repeated three times. The effects of different injection rates (200, 300, 400, 500, and 600 ml/hr) on the preparation of microspheres were investigated.

### 2.7 Orthogonal design optimization experiment of microsphere preparation process

According to the results of single factor investigation, the main factors affecting the quality of microspheres were chitosan mass fraction (*a*), quality ratio of drug to chitosan (*b*), inlet air temperature (*c*), and injection rate (*d*). The “(DL + YD)/2” was used as the comprehensive evaluation index, and the L_9_ (3^4^) orthogonal design was used to optimize the microsphere preparation process and prescription. The orthogonal experimental parameters were shown in Table 2.

**Table 2:** Orthogonal experimental parameters

Level	Test factor			
	*a* (%)	*b* (g/g)	*c* (°C)	*d* (ml/hr)
1	1	1:2	120	300
2	1.5	1:3	130	400
3	2	1:4	140	500

### 2.8 Particle size

An appropriate amount of chitosan‐based drug microspheres was diluted with water, and the particle size was then measured using BT‐9300H Laser Particle Size Analyzer.

### 2.9 SEM

The dried chitosan‐based drug microspheres were fixed on the sample stage with a conductive double‐sided adhesive. The gold‐sprayed samples were placed in a KYKY‐EM6200 scanning electron microscope to observe the surface morphology of the microspheres and take pictures of their morphology.

### 2.10 Release assay in vitro

The experiment was carried out with 900 ml of phosphate buffer (pH = 4.5) as the release medium at a temperature of (37 ± 0.5)°C and a rotation speed of 100 r/min (Desai, 2010). Precisely weigh 20 mg of drug powder and drug‐loaded chitosan microspheres into a large cup, and took out 5 ml at regular intervals (1, 2, 3, 4, 5, 6, 7, 8, 9, 10, 11, and 12 hr), and timely replenished the release medium of the same volume and temperature. The sample was filtered through a micropore filter, and the concentration of notoginsenoside R1 was determined by HPLC. At the same time, the cumulative release rate in vitro was calculated.

### 2.11 Statistical analysis

All experiments were performed in triplicate, and a completely randomized design was used. Data are expressed as mean ± standard error of the mean (*SEM*).

## 3 RESULTS

### 3.1 Single factor experiment results of microsphere preparation process

#### 3.1.1 Chitosan mass fraction

**Figure 1:**
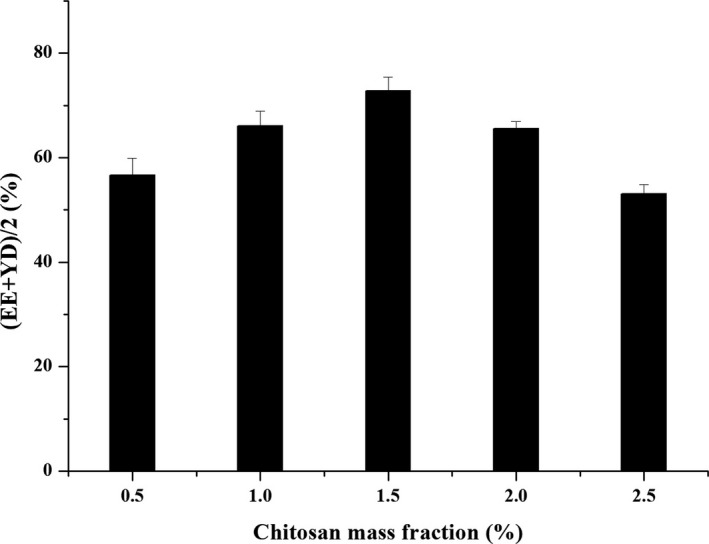
The effect of chitosan mass fraction on (EE + YD)/2

As shown in Figure 1, the comprehensive evaluation index of drug loading and yield increased with the increase of chitosan mass fraction, and maximum value was achieved when the mass fraction of chitosan oligosaccharide was 1.5%. However, when the chitosan mass fraction continued to increase, the comprehensive index was reduced due to the serious sticking phenomenon and poor atomization effect. Therefore, the optimum mass fraction of chitosan oligosaccharides was selected as 1.5%. The experimental results were in agreement with the actual results of the measured values of solution viscosity.

#### 3.1.2 Mass ratio of drug to chitosan

**Figure 2:**
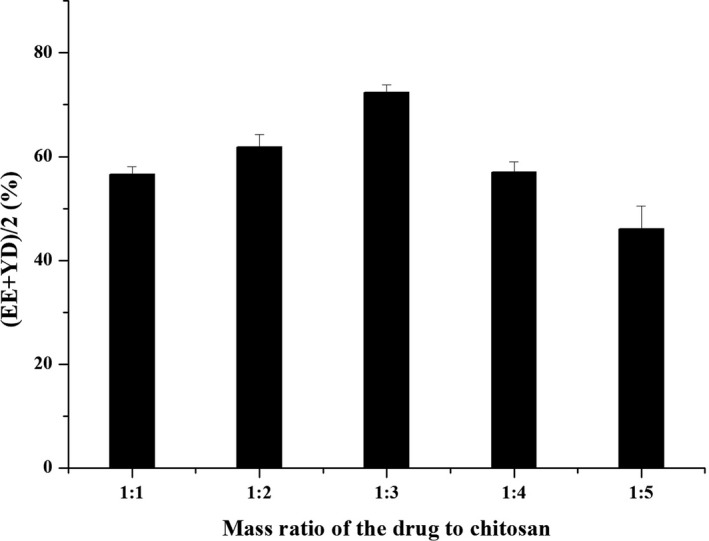
The effect of the mass ratio of drug to chitosan on (EE + YD)/2

As shown in Figure 2, the comprehensive evaluation index of drug loading and yield increased with the increase of the mass ratio of drug to chitosan, and maximum value was achieved when the mass ratio was 1:3. However, as the mass ratio continued to increase, the encapsulation rate showed a downward trend, which made the overall index show a downward trend. Therefore, the optimum mass ratio of the extract of chitosan was selected as 1:3.

#### 3.1.3 Inlet air temperature

**Figure 3:**
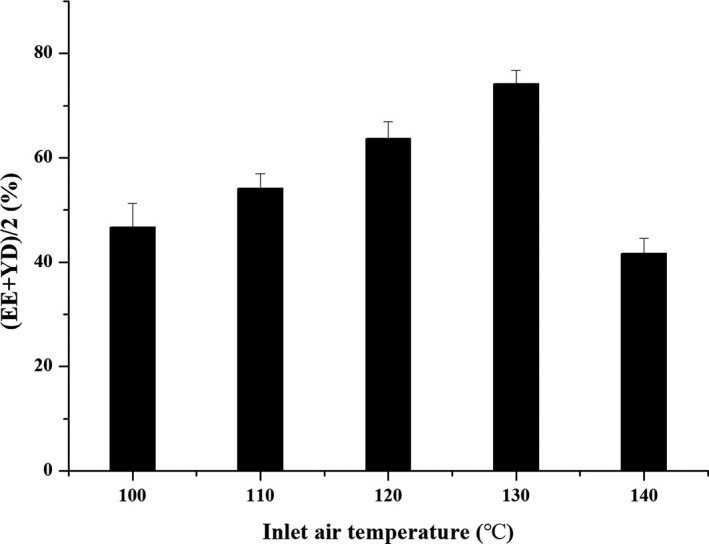
The effect of inlet air temperature on (EE + YD)/2

As shown in Figure 3, the inlet air temperature was closely related to the comprehensive evaluation index of drug loading and yield. The comprehensive evaluation index gradually increased with the increase of the inlet air temperature and reached maximum value at 130°C. Subsequent increase in temperature would cause the microspheres disperse quickly, making the microspheres appear concave and easily broken, and affect the quality of the microspheres, which was detrimental to the final yield and drug loading. Therefore, the optimum inlet air temperature was selected as 130°C.

#### 3.1.4 Injection rate

**Figure 4:**
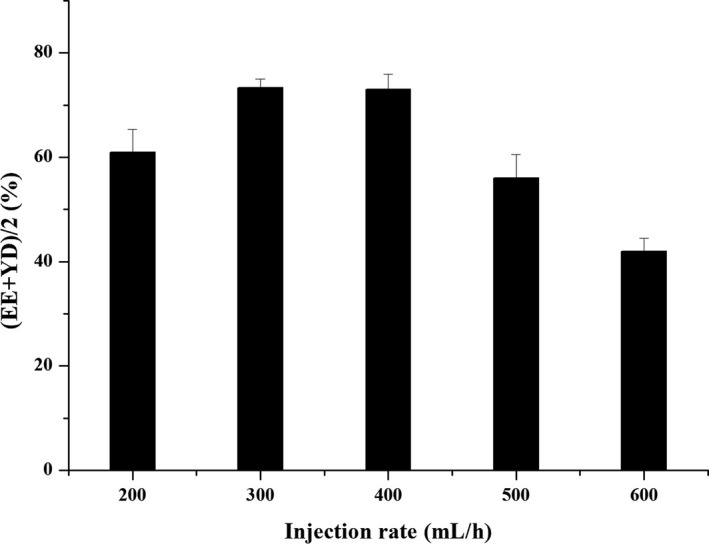
The effect of injection rate on (EE + YD)/2

As shown in Figure 4, the comprehensive evaluation index of drug loading and yield showed an increasing trend with the increase of injection rate until 400 ml/hr. When the injection rates of 300 and 400 ml/hr were selected, the comprehensive evaluation indexes of the microspheres were not much different. However, when it exceeded 400 ml/hr, the microspheres were difficult to form. This result was due to many factors such as sticky wall and poor evaporation effect, which might result in excessive particle size and poor drying effect and a decrease in the overall index. Considering the efficiency and productivity of spray drying, injection rate of 400 ml/hr was appropriate.

### 3.2 The experimental results of the orthogonal design of microsphere preparation process

According to the orthogonal test factors and the level table in Table 2, the orthogonal design and the results were shown in Table 3 below:

**Table 3:** Orthogonal test design and results

Test number	*a*	*b*	*c*	*d*	Comprehensive indicator
1	1	1	1	1	74.61
2	1	2	2	2	78.48
3	1	3	3	3	70.09
4	2	1	2	3	72.51
5	2	2	3	1	68.37
6	2	3	1	2	69.35
7	3	1	3	2	71.82
8	3	2	1	3	73.13
9	3	3	2	1	71.76
K_1_	74.393	72.980	72.363	71.580	
K_2_	70.077	73.327	74.250	73.217	
K_3_	72.237	70.400	70.093	71.910	
K_4_	4.316	2.927	4.157	1.637	

The effect of the factors was chitosan mass fraction, inlet air temperature, mass ratio of drug to chitosan, injection rate, in turn. From the results, the best process and prescription from a single factor experiment (*a*_2_ *b*_2_ *c*_2_ *d*_2_) and the orthogonal test (*a*_1_ *b*_2_ *c*_2_ *d*_2_) were close to each other. However, considering that the higher the ratio of the capsule material, the better the encapsulation effect was. The optimal preparation process and prescription for the microspheres were finalized as *a*_2_ *b*_2_ *c*_2_ *d*_2_. The mass fraction of chitosan was 1.5%, the mass ratio of drug to chitosan was 1:3, the inlet air temperature was 130°C, and the injection rate was 400 ml/hr.

### 3.3 Verification test

Three batches of chitosan‐based microspheres were prepared according to the optimal process and formulation conditions obtained from the above orthogonal test results. The EE, DL, and YD of the microspheres were 91.28 ± 1.04%, 23.87 ± 0.93%, and 70.92 ± 0.81%, respectively, indicating that the reproducibility of the preparation process was good.

### 3.4 Particle size

The average particle size of the drug powder and microspheres was shown in Table 4. The particle size of the drug powder and Chitosan‐based drug microspheres was 2.17 ± 1.08 and 10.27 ± 1.05 μm, respectively. It can be seen that the particle size of the drug powder was significantly increased after being coated with chitosan and prepared into microspheres by spray drying. This showed that the surface of the drug powder was covered with chitosan. The obtained microsphere sample was a whitish pale yellow powder with no bad odor and had good dispersibility and stability.

**Table 4:** Average particle size of drug powder and microspheres

	Drug power	Chitosan‐based drug microspheres
Particle size (*d*, μm)	2.17 ± 1.08	10.27 ± 1.05

### 3.5 SEM

**Figure 5:**
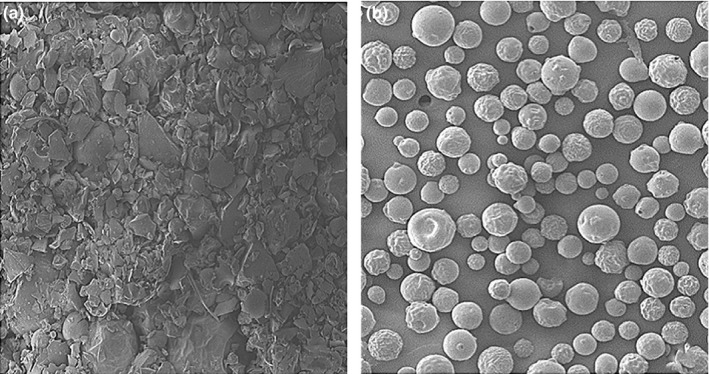
Drug powder samples (a) and microsphere samples (b) under SEM

As shown in Figure 5, the drug powder observed by SEM was irregular in size and rough in surface. However, after the drug powder was embedded in chitosan and spray‐dried into microspheres, the microspheres observed by SEM had a round shape, a smooth surface, and no adhesion. Further shows that the surface of the drug powder is fully covered with chitosan.

### 3.6 Release assay in vitro

**Figure 6:**
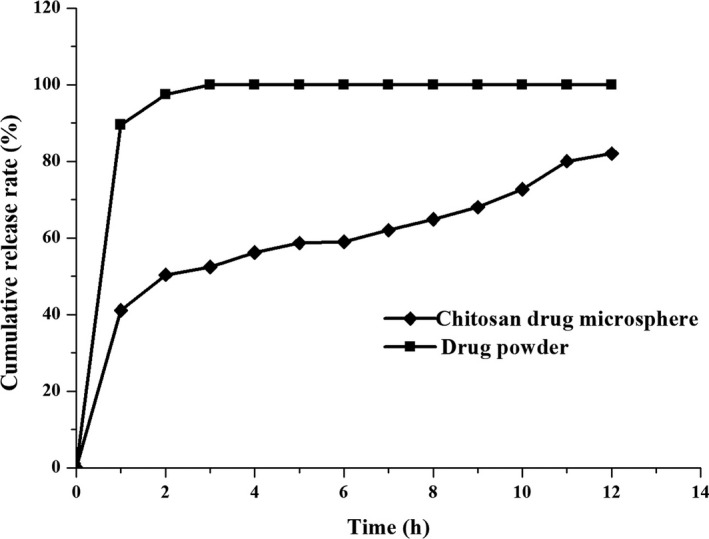
The release curve of drug powder samples and microsphere samples

Taking the drug powder as a control, the results were shown in Figure 6. It can be seen that the chitosan‐based microspheres had a significant sustained release effect. The release behavior of the microspheres in vitro was divided into two stages: The release rate of the drug in the previous stage was faster, and it was the release phase, which accumulated 41.08% within 1 hr; the release rate of the drug in the latter stage became slower and showed a significant sustained release. The release curve was fitted to the Higuchi model, and the fitted equation of the sample was *Q* = 14.99 *t* ^1/2^ + 25.729, *r* = .9701. The early burst release was beneficial to the drug to reach a higher concentration in the stomach for a short time. The smooth release of the follow‐up drug was beneficial to maintain the effective concentration for a long time.

## 4 DISCUSSION

1. In this study, the chitosan‐based microspheres prepared by spray drying, whose surface was smooth, round, and non‐adhesive, and drug loading reached 23.87%. The method of spray drying has the advantages of convenient operation, easy control of product quality, without organic solvent during the preparation process, and is suitable for industrial production and the like. This study will provide experimental references for the development of chitosan‐based microsphere solid preparations.
2. In this study, phosphate buffer (pH = 4.5) was used as the release medium in vitro. The results showed that the release rate of microspheres reached 41.08% within 1 hr from the start, and there was a burst effect. On the one hand, this may be caused by the rapid dissolution of tiny drug particles attached to the surface of the microspheres. On the other hand, the release medium rapidly enters the inside of the microspheres through the micropores and dissolves the drug. The release rate within 2–12 hr is relatively slow. This is because that chitosan is easy to absorb water and swells. The swelling behavior of chitosan reduces or even eliminates the micropore diameter, causing the micropore channel close. Therefore, the subsequent drug release can only slowly spread through the skeleton, or slowly released by the degradation of chitosan.
3. The measurement conditions of the release test in vitro are difficult to simulate the biological environment of human body. Therefore, the release in vitro is mainly used as an indicator for the screening of prescriptions. The specific drug absorption and the actual effects in vivo need to be further studied by absorption experiments in vivo.

## 5 CONCLUSION

The chitosan‐based release microspheres were prepared by spray drying method. Chitosan was used as the carrier material, and *P. notoginseng* extract, *Codonopsis* extract, and *Atractylodes* extract (the mass ratio was 2:7:5) were active substance. The mass fraction of chitosan was 1.5%, the mass ratio of drug to chitosan was 1:3, the inlet air temperature was 130°C, and the injection rate was 400 ml/hr. The chitosan‐based microspheres prepared under the above conditions had a smooth surface, and the DL was 23.87 ± 0.93%; the average particle diameter was 10.27 ± 1.05 μm, and the EE of the microspheres was 91.28 ± 1.04%. The preparation process of chitosan‐based drug microsphere prepared by spray drying method was simple and stable. The prepared microspheres in this paper showed a sustained release effect in vitro. This study will provide experimental references for the development of chitosan‐based microsphere solid preparations.

## CONFLICT OF INTEREST

The authors declare no conflict of interests.

## ETHICAL APPROVAL

This article does not contain any studies with human participants or animals performed by any of the authors.
